# Metagenomic Analysis Reveals Viral Diversity in Phlebotomine Sand Flies from Caribbean Region in Colombia

**DOI:** 10.3390/microorganisms14061343

**Published:** 2026-06-15

**Authors:** Luis Romero-Ricardo, Yesica López, Yeimi Lopez-Mejia, Alejandra García, Héctor Contreras-Martínez, Ketty Galeano, Bertha Gastelbondo, Pedro Fragoso, Luis Paternina, German Arrieta, Salim Mattar

**Affiliations:** 1 Facultad de Medicina Veterinaria y Zootecnia, Instituto de Investigaciones Biológicas del Trópico, Universidad de Córdoba, Montería 232520, Córdoba, Colombia; lr.romero.ricardo@gmail.com (L.R.-R.); yesicalopezm@correo.unicordoba.edu.co (Y.L.); yeimilopezm@correo.unicordoba.edu.co (Y.L.-M.); agarciaperez@correo.unicordoba.edu.co (A.G.); hectorcontrerasm@correo.unicordoba.edu.co (H.C.-M.); kettygaleanoa@correo.unicordoba.edu.co (K.G.); berthagastelbondop@correo.unicordoba.edu.co (B.G.); germanarrieta@correo.unicordoba.edu.co (G.A.); 2 Departamento de Medicina Tropical, Facultad de Medicina, Universidad de Cartagena, Cartagena 130001, Bolívar, Colombia; 3 Grupo de Investigación Parasitología y Agroecología Milenio, Facultad Ciencias de la Salud, Universidad Popular del Cesar, Valledupar 200004, Cesar, Colombia; pedrofragozo@unicesar.edu.co; 4 Departamento de Biología y Química, Facultad de Educación y Ciencias, Universidad de Sucre, Sincelejo 700001, Sucre, Colombia; luis.paternina@unisucre.edu.co; 5 Grupo de Salud Pública y Auditoría En Salud, Facultad de Ciencias Económicas y Administrativas, Corporación Universitaria del Caribe (CECAR), Sincelejo 700001, Sucre, Colombia

**Keywords:** sand fly, virome, *Lutzomyia gomezi*, *Pintomyia evansi*, *Rhabdoviridae*, *Dicistroviridae*, Phenuiviridae

## Abstract

Phlebotomine sand flies are dipterans that transmit leishmaniasis, bartonellosis, and arboviruses of public health importance. Colombia is a tropical country with high annual incidences of arboviruses, such as dengue and, more recently, yellow fever, all of which have similar symptoms. This study characterized the viruses circulating in phlebotomine sand flies in two departments in the Colombian Caribbean. Between August 2023 and December 2024, a descriptive study was conducted in the Departments of Córdoba and Cesar in Colombia. Four municipalities were selected per department, and four insect captures were performed using CDC light traps. Specimens were taxonomically identified and organized into groups according to species and study area, and total RNA was extracted for NGS analysis. Short sequences were quality-assessed, assembled using MEGAHIT to obtain contigs, and classified using DIAMOND-MEGAN6 to select viral genomic sequences for phylogenetic analysis. Thirteen viral families were identified, including a virus from the family *Rhabdoviridae* in *Pi. evansi* in the department of Cesar and another from the family *Dicistroviridae* in *Lutzomyia gomezi* in both departments. Two genome segments of the family *Phenuiviridae* were found in *Lutzomyia gomezi* in the department of Córdoba, Colombia. Sand flies harbor a diverse range of viral families, some of which are previously undescribed, and can be studied to determine their taxonomy and assess their potential to infect vertebrate cells or their interactions with medically important pathogens such as *Leishmania* spp.

## 1. Introduction

Arboviruses are a group of viruses transmitted by arthropods. These infections represent a global public health concern, with dengue, Zika, accounting for more than 13 million cases in the Americas, according to the Pan American Health Organization (PAHO) [1]. The recent resurgence of yellow fever has renewed public health concern in several endemic regions [2]. Both viruses are primarily transmitted by mosquitoes; however, other viruses can also cause serious health problems in humans and are transmitted by other arthropod vectors, such as ticks of the family Ixodidae, flies of the family Ceratopogonidae, and Diptera of the subfamily Phlebotominae [3].

Advances in metagenomic approaches based on RNA sequencing (RNA-seq) have improved our understanding of the viral diversity present in medically important arthropods, including mosquitoes [4,5], phlebotomine sand flies, ticks [6] and triatomine bugs [7]. These studies have contributed to the discovery of new viruses, the surveillance of specific viral groups [8] and the characterization of microbial communities associated with arboviruses [9]. In addition, metagenomics provides baseline information for the detection and characterization of emerging pathogens, which may be useful when investigating febrile illnesses of unknown origin, particularly in regions where several vector-borne viral diseases coexist and continue to represent a public health challenge [8,10].

Phlebotomine sand flies (Psychodidae: Phlebotominae) are implicated in the transmission of a wide range of pathogens, including parasites, bacteria, and viruses [11,12]. Viruses belonging to the families *Phenuiviridae*, *Rhabdoviridae*, and *Reoviridae* are important for public health. The genus *Phlebovirus* (*Hareavirales*: *Phenuiviridae*) is of public health importance because it can cause fever, myalgia, weakness, headache, and retro-orbital pain in humans. Some viruses affect the central nervous system, leading to depression, meningitis, and severe neurological manifestations [13,14,15]. These symptoms are nonspecific and may overlap with those caused by dengue virus infections and other arboviral diseases [16].

In recent years, metagenomic approaches have considerably improved the characterization of viral communities associated with phlebotomine sand flies, contributing to both epidemiological surveillance and the discovery of new viruses [17]. However, most of these studies have been conducted in Europe and Asia, and information available for the Americas remains scarce. To date, only limited metagenomic research has been conducted in the region, including the description of *Phlebovirus pantanalense* in Brazil [18] and detection of some viruses [19], highlighting the need to expand studies on viral diversity associated with sand flies in the Neotropical region. In Colombia little is known about the sand fly-born viruses, nonetheless, the detection of pathogenic viruses in the Colombian Caribbean [20] and the recent identification of phleboviruses in the department of Sucre demonstrate the importance of studying the sand fly virome in this region [21].

This study aimed to characterize the sand fly virome in two departments of the Colombian Caribbean.

## 2. Materials and Methods

### 2.1. Capture and Identification of Sand Flies

**Figure 1:**
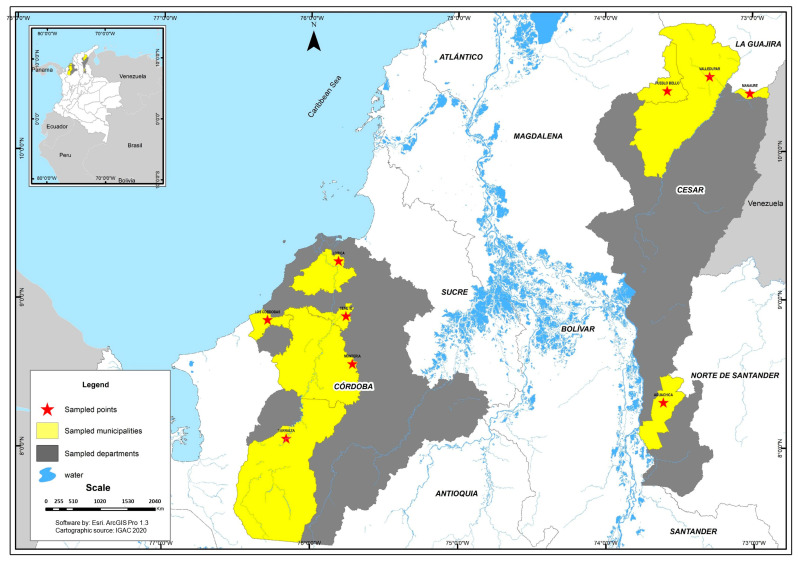
Geographic distribution of the sand fly capture sites in the departments of Córdoba and Cesar.

Between August 2023 and December 2024, two captures were conducted: one during the rainy season and the other during the dry season. Insect samples were taken in the municipalities of Cereté, Los Córdobas, Lorica, Montería, and Tierralta in the department of Córdoba, and in the municipalities of Valledupar, Manaure, Pueblo Bello, and Aguachica in the department of Cesar (Figure 1). CDC light traps were operated from 18:00 to 06:00 h the following day. Sand flies were identified using taxonomic keys [22]. The samples were separated and grouped into up to 70 individuals depending on species, sex, location, and dry or rainy period.

### 2.2. Library Preparation and RNA NGS

Sand fly groups were macerated in 300 µL of 1X PBS, and 150 µL was used for RNA extraction. The homogenate was filtered through a 0.45 µm filter for total RNA extraction using the GeneJET Viral DNA/RNA Purification Kit (Waltham, MA, USA). The RNA obtained was quantified using a Qubit 4 flex spectrofluorometer, and values < 10 ng were discarded. RNA from the same species in different municipalities in each department was combined. RNA from the samples was subsequently fragmented, and first- and second-strand cDNA was synthesized. Library preparation was performed following the manufacturer’s recommendations using the MGIEasy RNA Library Prep Kit V3.0 (Yantian District, Shenzhen, China). Finally, the prepared DNBs were placed in a DNBSEQ-G50RS Sequencing Flow Cell and sequenced in paired-end format on the DNBSEQ-50 for 32 h.

### 2.3. Bioinformatics Analysis

Read quality was verified using fastp software v.1.3.3 [23]. Considering the variable nature of viral nucleotide sequences, this study employed a pipeline focused on the characterization of viral protein-coding transcripts. This protocol consisted of de novo assembly to obtain RNA transcripts using MEGAHIT v.1.2.9 [24] and taxonomic assignment using DIAMOND-MEGAN6 [25], using a complete database downloaded from GENBANK in September 2023. Transcripts identified as viral were cross-referenced with the GenBank database using the online BLASTx tool v.2.16.0 [26]. Subsequently, some of the complete viral genome assemblies were selected and subjected to NCBI’s ORF Finder to identify potential ORFs in the genome. The obtained sequences were annotated and translated using Prokka v.1.14.6 on the Galaxy online platform (https://galaxy-main.usegalaxy.org, accessed on 12 May 2025) [27,28]. After Prokka translation, the central domain of the RNA-dependent RNA polymerase (RdRp) was selected to infer the phylogenetic relationships among the different viral groups. The sequences corresponding to the predicted ORFs were translated and compared with the NCBI non-redundant (nr) protein database using BLASTp v.2.16.0. Additionally, a search was conducted for conserved protein domains to complement and support the putative functional assignment of identified viral proteins.

The obtained amino acid sequences were aligned with reference sequences retrieved from GenBank [29] using the Multiple Alignment using Fast Fourier Transform (MAFFT) v.7.526 [30]. Subsequently, the relationship between the obtained sequence and the reference sequences was estimated using an identity matrix, using the Sequence Demarcation Tool (SDT) v.1.3 [31], and by determining phylogenetic relationships based on Maximum Likelihood (ML) in IQTREE (http://iqtree.cibiv.univie.ac.at/, accessed on 12 May 2025), using 2000 ultrafast bootstraps [32]. Finally, the trees were visualized and edited using iTOL v.5 [33]. The depth of the sequence assemblies was determined by mapping raw reads of the complete or partial viral genomes using Bowtie2 v.2.5.3 [34], and the results were visualized using UGENE v.50.0 [35].

## 3. Results

### 3.1. Captured Sand Flies

A total of 3010 sand flies were collected and grouped into 16 species; 83.6% of the individuals were captured in the department of Córdoba. The three most prevalent sand fly species in both departments were *Lutzomyia* (*Lu*.) *gomezi* 1116/3010 (37%), *Psathyromyia* (*Pa.*) *carpenteri* 544/3010 (18%), and *Bichromomyia* (*Bi*) *olmeca bicolor* 507/3009 (16.8%) (Table 1). In the department of Córdoba, *Bi. olmeca bicolor* was found for the first time. In the department of Cesar, *Lu. lichyi*, *Helcocyrtomyia* sp., *Pa. barrettoi majuscula*, *Pa. shannoni*, *Psychodopygus* (*Ps.*) *panamensis* and *Pa. carpenteri* were recorded for the first time in the country.

**Figure 2:**
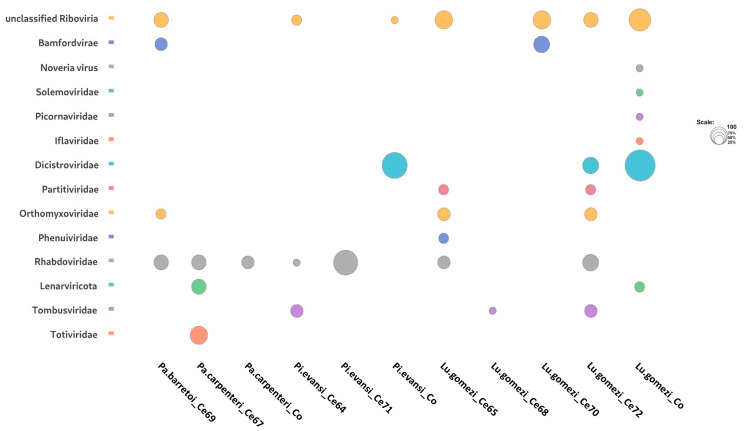
Comparative taxonomic profile and frequency of viral families by sand fly species. The size of the circles represents the relative abundance of the contigs assigned to each viral taxon in each sample. Larger bubbles indicate a higher proportion of contigs belonging to a specific viral group relative to the total viral sequences detected in the analyzed samples.

Fifteen groups were selected based on the location, quantity and quality of their genetic material (Table 2). Sequencing revealed that 11 of the 15 sequenced samples contained viral genetic material. The assembly yielded transcripts belonging to viral families *Phenuiviridae*, *Rhabdoviridae*, *Dicistroviridae*, *Bamfordvirae*, *Solemoviridae*, *Picornaviridae*, *Iflaviridae*, *Partitiviridae*, and *Orthomyxoviridae*. *Lu. gomezi* exhibited the highest viral richness, with more than five viral families present. *Rhabdoviridae* was the most frequent family, detected in six sand fly groups from the department of Cesar and one group from the department of Córdoba (Figure 2).

### 3.2. Genomic Characterization of Viruses in Sand Flies

**Figure 3:**
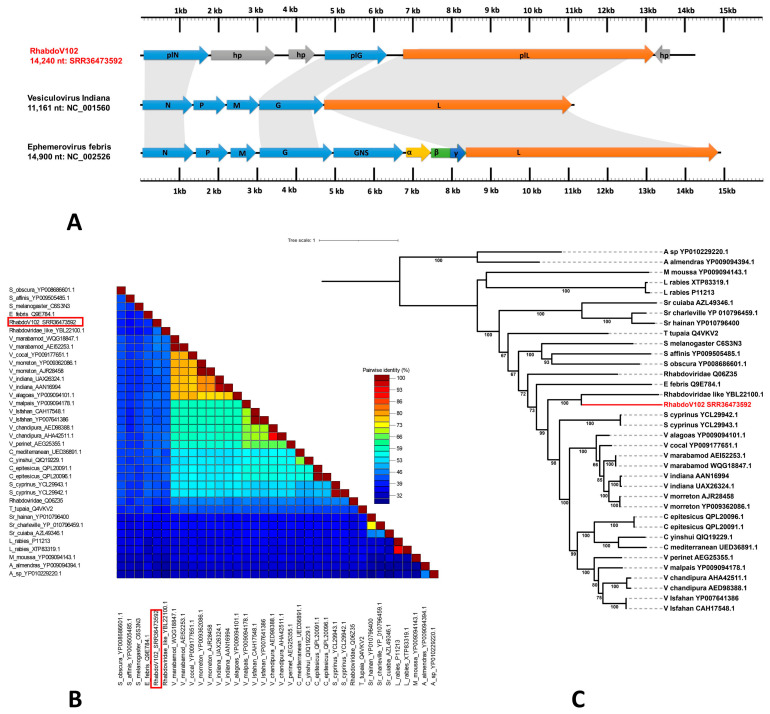
Genetic characterization of assembled rhabdoviral sequence (highlighted in red). Comparison of the obtained genomic sequence with two reference sequences from the family *Rhabdoviridae*. Gray arrows indicate the hypothetical gene obtained during the genomic annotation process. The orange arrows indicate the genes used for the genetic characterization of the obtained virus. Blue arrows indicate common genes found in rhabdoviruses. Gray areas correspond to similar amino acid sequences. Glycoprotein (G); Nucleoprotein (N); Phosphoprotein (P); Matrix protein (M); Non-structural glycoprotein (GNS); L polymerase (L); Protein-like Nucleoprotein (plN); protein-like Glycoprotein (plG); protein-like L polymerase (plL); hypothetical protein (hp) (**A**). Pairwise nucleotide identity matrix (**B**). Maximum likelihood (ML)-based phylogenetic inference of the amino acid sequences of L gene of the family *Rhabdoviridae*, applying the Q.pfam+F+I+R6 evolutionary model and 2000 bootstrap replicates (**C**).

In the family *Rhabdoviridae*, an assembled segment of 14,240 nucleotides was obtained in a pool of *Pi. evansi* from the department of Cesar (SRR36473592, Dataset S1). The five predicted coding regions included ORFs that showed similarity to rhabdovirus nucleoproteins (pIN), glycoproteins (pIG), and RNA-dependent RNA polymerases (RdRp) (pIL), based on amino acid similarity searches and conserved domain analysis. The largest ORF comprised 6350 nucleotides and encoded a putative RdRp protein of 2116 amino acids (Figure 3A). Additionally, two intermediate ORFs were identified between the putative nucleoprotein and glycoprotein-encoding genes. Based on their genomic position and predicted length of the encoded proteins, these ORFs could correspond to highly divergent genes similar to phosphoprotein (P) and matrix protein (M), typically present in rhabdoviruses. However, searches performed using BLASTp did not detect significant similarities with sequences available in the databases; therefore, both ORFs were conservatively annotated as hypothetical proteins.

However, the recovery of continuous coding sequences and their location within the genomic region normally occupied by accessory proteins in rhabdoviruses supports their classification as genuine viral ORFs. Furthermore, their amino acid compositions and genomic contexts are consistent with those of highly divergent viral proteins, although their specific functions have not yet been determined.

Regarding the relationship between our assembled sequence and reference sequences from various genera, the identity matrix showed a percentage greater than 46% with the sequence YBL22100.1, which corresponds to an unclassified rhabdovirus, while it had identity values equal to or less than 40% with other sequences from the *Rhabdoviridae* family (Figure 3B). The phylogenetic analysis was conducted with the ORF pIL. The sequence was aligned with 35 sequences of RdRp complete gene from the family *Rhabdoviridae* available in GenBank with up to 2329 amino acids. The alignment allowed the identification of 1850 parsimoniously informative sites and 268 conserved sites. These results, together with phylogenetic inference, confirmed its relationship with the family *Rhabdoviridae*, forming a clade independent of the unclassified sequence Rhabdoviridae-like, obtained from sand fly in a previous work (Figure 3C).

**Figure 4:**
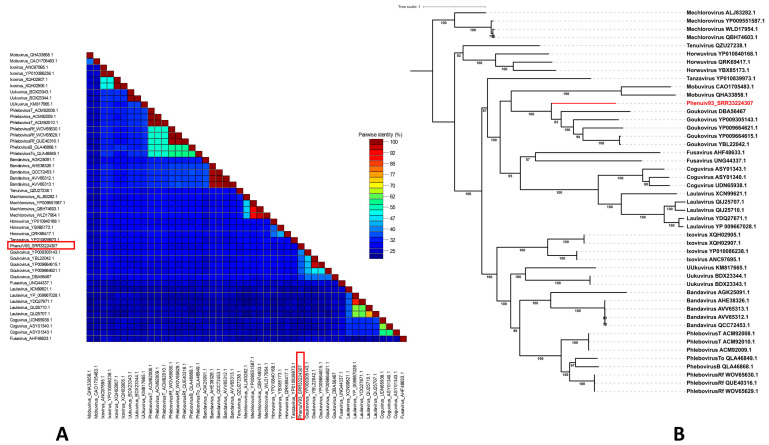
Genetic characterization of assembled phenuivirus sequence (highlighted in red). Pairwise nucleotide identity matrix (**A**). Maximum likelihood (ML)-based phylogenetic inference of the amino acid sequences of the family *Phenuiviridae*, applying the LG+F+I+G4 evolutionary model and 2000 bootstrap replicates. The sequence obtained in the study is highlighted in red (**B**).

A 3151-base genome assembly was found for the family *Phenuiviridae*, compatible with the M segment (SRR33224308, Dataset S1), and a 6648-base genome assembly was found to be related to the L segment (SRR33224307, Dataset S1). When evaluating the L segment as a region for molecular taxonomy, the presence of the DUF3770 domain, N-terminal domain of the L protein, and central domain of RdRp were determined, with a total of 2130 amino acids. This latter segment was aligned with 46 reference sequences, with 2893 parsimoniously informative sites and 404 conserved sites. The percentages of amino acid sequence identity when comparing our assembly with other sequences were greater than 40% with sequences from the genus *Goukovirus*, while they were lower with other sequences from the family *Phenuiviridae.* These values were confirmed by phylogenetic inference, forming a highly supported monophyletic clade between our contig (PhenuiV93_SRR33224307), found in a group of *Lu. gomezi*, and individuals of the genus *Goukovirus* (Figure 4B).

**Figure 5:**
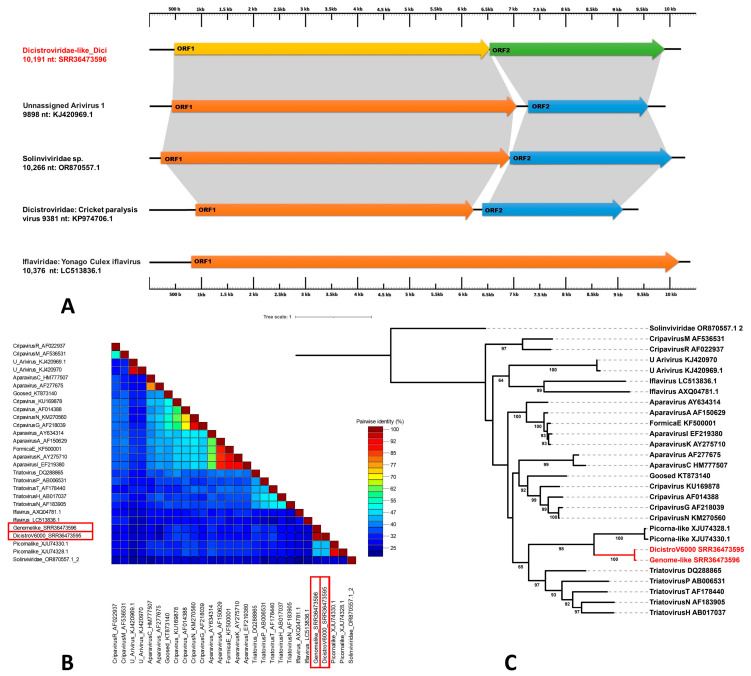
Genetic characterization of the assembled picornaviral sequences (highlighted in red). Comparison between the obtained genomic sequence and some *Dicistroviridae* genera. Non-structural proteins (ORF1) and structural proteins (ORF2) (**A**). Pairwise nucleotide identity matrix (**B**). Phylogenetic inference based on the Maximum Likelihood (ML) of amino acid sequences with 456 positions of the viral polymerase domain of families of the order *Picornavirales* (through BMGE selection), applying the LG+F+I+G4 evolutionary model and 2000 bootstrap replicates (**C**).

Additionally, sequences from the family *Dicistroviridae* were identified in *Lu. gomezi* and *Pi. evansi* from Córdoba, and in *Lu. gomezi* from Cesar. It is important to highlight the 10,191-nucleotide assembly identified in a pool of *Pi. evansi* in Córdoba (SRR36473596). The genome structure was consistent with that of other members of the family (Figure 5A), including an ORF of 6240 nucleotides that encodes a polyprotein containing an RNA helicase, protease, and RdRp. The second ORF (ORF2), spanning 3219 nucleotides, encodes a polyprotein of 1723 amino acids comprising the three major structural proteins. Due to the high variable regions and gaps, the RdRp region in ORF1 was selected by clipping the segment of interest identified by the blastp “Conserved Domains”. Alignment with the reference sequences allowed the analysis of 398 amino acid positions, of which 296 were informative according to parsimony and 61 were conserved.

Due to the low support for the complete genome of the *Dicistroviridae* virus, other shorter sequences (2000, 3000 and 6000 nucleotides) obtained from the same *Pi. evansi* pool were subjected to mapping tests (SRR36473593, SRR36473594, SRR36473595, Dataset S1). These sequences were supported by high read coverage and showed high sequence similarity with the assembled genome (10,191 nucleotides), supporting the recovery of a Dicistroviridae-like genome from the analyzed sample.

The sequences SRR36473595 and SRR36473596 assembled in this study shared more than 90% nucleotide identity with each other. In contrast, they showed only 47% identity to two unclassified picornavirus sequences and less than 40% identity to all other sequences included in the analysis (Figure 5B). Consistent with these results, phylogenetic reconstruction grouped both sequences within a well-supported monophyletic clade together with the unclassified picornaviruses, positioned closest to members of the *Triatovirus* genus (Figure 5C).

## 4. Discussion

The results of this study documents additional records of sand flies in the departments of Córdoba and Cesar and reveal 13 previously unreported viral families. The virome highlights genomic segments from the *Dicistroviridae*, *Rhabdoviridae*, and *Phenuiviridae* families in sand flies *Pi. evansi* (formerly *Lu. evansi*) and *Lu. gomezi*, both of which are known for their anthropophilic behavior [36,37].

In the Cesar department, a new species report was obtained, including *Lu. lichyi*, *Helcocyrtomyia* sp., *Pa. barrettoi majuscula*, *Pa. shannoni*, *Ps. panamensis* and *Pa. carpenteri* were identified [38]. It is important to note that the *Ps. panamensis*, *Helcocyrtomyia* sp., *Lu. lichyi*, and *Pa. shannoni* are implicated in the transmission of *Leishmania* spp. to humans [11,39,40]. In addition, the presence of *Bi. olmeca bicolor*, an anthropophilic sand fly considered a vector, was recorded for the first time in the department of Córdoba [39]. These findings, together with the anthropophilic habits and the presence of other well-known vectors in these two departments [41,42], highlight the potential for virus circulation in these areas.

This study represents the first metagenomic survey of viruses in sand flies from Colombia and identified members of the families *Rhabdoviridae*, *Phenuiviridae*, and *Dicistroviridae*. We detected *Rhabdoviridae* transcripts in most of the pools we analyzed. When we first classified the assembled contigs, several matched members of the genus *Vesiculovirus*. That group is diverse and includes several viruses that matter for public health [43,44,45].

However, when we ran the phylogenetic analysis on the largest rhabdovirus-like genome (about 14 kb), it showed a different relationship. Instead of clustering with known vesiculoviruses, this sequence grouped with an unclassified rhabdovirus-like genome from public databases. Interestingly, that sequence was also obtained from a pool of sand flies from the state of Pará, Brazil [19]. Although the available evidence is insufficient for a more precise taxonomic assignment, the association of both sequences with phlebotomine sand flies may indicate the presence of closely related viruses in these insects across geographically distant regions.

The recovery of a nearly complete rhabdovirus-like genome from sand flies expands our knowledge of the viral diversity associated with these insects. The genome exhibited the characteristic organization of members of the *Rhabdoviridae* family, and three of its predicted ORFs were associated with N, G, and L genes. The presence of the conserved Rhabdo_ncap domain in the putative nucleoprotein [46], along with the significant similarity of the glycoprotein and polymerase to those of previously recognized rhabdoviruses, strongly supports the viral nature of the assembled sequence.

In addition to the conserved genes, two intermediate ORFs sit between the putative N and G genes. BLASTp searches turned up no significant matches to any proteins in public databases. Still, their location in the genome and their coding potential suggest they are likely highly divergent viral genes. Other arthropod-associated rhabdoviruses carry similar ORFs. In those cases, accessory or lineage-specific proteins often share little or no detectable similarity with known sequences [47,48]. The data available here do not allow us to determine what these ORFs do biologically. But given where they are positioned—inside a genome that otherwise has the typical rhabdovirus layout—it seems unlikely they are just random coding regions. The lack of detectable homologs probably reflects how genetically diverse arthropod-associated rhabdoviruses really are, and how poorly these viruses are still represented in current databases. Sorting out what these predicted proteins actually do will take more work, especially virus isolation and experimental follow-up.

The genomic complexity of rhabdoviruses may be explained by the loss and acquisition of new ORFs, known as accessory genes. Some of these genes lack amino acid homology when translated and compared with reference sequences and are therefore referred to as orphan proteins [49,50]. Although some predicted proteins showed relatively low amino acid identity values compared to currently available reference sequences, the conserved domains and genomic organization were consistent with those reported for members of the *Rhabdoviridae* family. These low similarity values are frequently observed in highly divergent arthropod-associated rhabdoviruses and could reflect the limited representation of related viruses in public databases rather than artifacts derived from the assembly process.

It is relevant to mention that the new virus identified here was found in *Pi. evansi* (Pi_evansi_CE64) from the municipality of Manaure, in the department of Cesar. This hematophagous sand fly species is of particular public health importance in the Caribbean region of Colombia because of its role in transmitting *Le. infantum* and *Le. braziliensis*, the causative agents of visceral and cutaneous leishmaniasis, respectively [51,52,53,54,55].

The family *Phenuiviridae* includes a wide range of viruses, including genera of public health importance, such as *Phlebovirus* and *Uukuvirus* [56,57]. In this study, we also detected insect-specific viruses (ISVs) of the genus *Goukovirus*, which has previously been described in *Culex declarator* mosquitoes in Trinidad [58], *Aedes aegypti* in China [59], and other *Anopheles* and *Uranotaenia* species from the Ivory Coast [60]. However, there are no previous reports of this viral genus in sand flies; thus, the present study is among the first to establish this association in the literature. ISVs can interfere with vectorial capacity [61], which is relevant in dipterans that transmit parasites of the *Leishmania* genus. Further research is needed to determine whether these viruses can modulate the transmission of pathogens in phlebotomine sand flies.

The assembled genome of the family *Dicistroviridae* presents an intergenic region (IGR) of only two nucleotides, a length that differs substantially from those typically reported in genera of this family, which range from 170 to 530 nucleotides [62]. This feature distinguishes the recovered genome from other described members of *Dicistroviridae* and suggests that the recovered virus may represent a distinct lineage within the family.

Phylogenetic analysis placed the assembled sequences together with two unclassified *Picornavirales*-like sequences that clustered within *Dicistroviridae* available in public databases. All four sequences formed a monophyletic clade and were recovered in pools of sand flies collected from Africa, published in Direct Submission by Entomology Laboratory, Walter Reed Army Institute of Research-Africa (XJU74328.1; XJU74330.1). The two reference sequences have not been classified beyond the family level. Although the available evidence is insufficient for a formal taxonomic assignment, the close relationship among these viruses suggests that they belong to a group that remains poorly characterized and underrepresented in current databases.

Among the classified members of the family,, the closest relatives of the assembled sequences were members of the genus *Triatovirus*, supporting their placement within the family *Dicistroviridae*. Members of this group have been reported in triatomines [63], other hemipterans [64,65], and bees [66]. To our knowledge, this is the first report of a virus belonging to this viral family in sand flies from the Americas. Together with the detection of members of the families *Rhabdoviridae*, *Flaviviridae*, *Phenuiviridae*, *Sedoreoviridae*, and *Peribunyaviridae* [67], these findings broaden current knowledge of the viral diversity associated with *P. evansi* in the department of Cesar.

The findings from this study serve as a foundation for future research and also provide new insights into the viral diversity associated with phlebotomine sand flies in Colombia. However, during the evaluation of the findings, there are some aspects that need to be considered. Further longitudinal studies are required to better understand the seasonality and ecological niches of these viruses in Diptera. The viral isolation and experimental characterization of the detected viruses were not performed. Therefore, the taxonomic assignment and putative function of some ORFs are based on evidence obtained through in silico analysis, including genomic organization, sequence similarity, and identification of conserved domains. Future studies incorporating viral isolation and molecular validation will allow for the confirmation of the predictions made. 

## 5. Conclusions

The detection of new viruses from the families *Dicistroviridae*, *Rhabdoviridae*, and *Phenuiviridae* highlights the remarkable viral diversity present in phlebotomine sand flies in the Colombian Caribbean and demonstrates that these insects harbor a broader range of viruses than has been reported in Colombia. Their detection in *Lu. gomezi* and *Pi. evansi* is relevant because both species frequently interact with humans and are involved in the transmission of parasites of the genus *Leishmania*. Some of the viral genomes recovered in this study were closely related to sequences previously reported from sand flies collected in other parts of South America and Africa. Although the available data do not allow conclusions about their ecological role, these findings suggest that related viruses may occur in phlebotomine populations from geographically distant regions.

## Figures and Tables

**Table 1:** Sand flies collected from the departments of Córdoba and Cesar, Caribe Colombia.

Species	Córdoba	Cesar	Individuals by Species
*Mi. atroclavata*	1	-	1
*Lu. lichyi*	-	1	1
*Pr. disponeta*	2	-	2
*Mg. migonei*	3	-	3
*Helcocyrtomyia* sp.	-	6	6
*Mi. micropyga*	8	-	8
*Pa. barrettoi majuscula*	-	8	8
*Pi. rangeliana*	9	2	11
*Ev. dubitans*	42	-	42
*Pa. shannoni*	7	107	114
*Pi. evansi*	87	73	160
*Ps. panamensis*	154	54	208
*Mi. cayennensis*	246	9	255
*Bi. olmeca bicolor*	507	-	507
*Pa. carpenteri*	521	23	544
*Lu. gomezi*	916	200	1116
*Brumptomyia* sp.	2	10	12
*Phlebotominae* sp.	12	-	12
Individuals by department	2517	492	3010

*Mi. Micropygomyia*; *Lu. Lutzomyia*; *Pr. Pressatia*; *Mg. Migonemyia*; *Pi. Pintomyia*; *Ev. Evandromyia*; *Pa. Psathyromyia*; *Bi. Bichromomyia*.

**Table 2:** Groups of sand flies evaluated for RNA sequencing, captured in different locations in the Departments of Córdoba and Cesar.

Pool ID	Sand Fly	Sex	Department	City
Lu_gomezi_CO	*Lu. gomezi*	Male and Female	Córdoba	Cereté, Montería, Los Córdobas, Tierra Alta
Pi_evansi_CO	*Pi. evansi*	Male and Female	Córdoba	Montería, Los Córdobas, Lorica
Pa_carpenteri_CO	*Pa. carpenteri*	Male and Female	Córdoba	Los Córdobas, Tierra Alta
Pi_evansi_CE64	*Pi. evansi*	Female	Cesar	Manaure
Lu_gomezi_CE65	*Lu. gomezi*	Male and Female	Cesar	Aguachica
Pa_carpenteri_CE67	*Pa. carpenteri*	Male	Cesar	Manaure
Lu_gomezi_CE68	*Lu. gomezi*	Male and Female	Cesar	Valledupar
Pa_barretoi_CE69	*Pa. barretoi majuscula*	Male and Female	Cesar	Pueblo Bello
Lu_gomezi_CE70	*Lu. gomezi*	Female	Cesar	Valledupar
Pi_evansi_CE71	*Pi. evansi*	Male and Female	Cesar	Valledupar
Lu_gomezi_CE72	*Lu. gomezi*	Male	Cesar	Valledupar

## Data Availability

Genomic segments and the corresponding sequenced reads mapped to them were submitted under the BioProject: PRJNA1253147 on the NCBI platform (https://dataview.ncbi.nlm.nih.gov/object/PRJNA1253147?reviewer=qeqdo8ogdk2f97avsm8ged6mof, accessed on 28 April 2026).

## Supplementary Materials

The following supporting information can be downloaded at: https://www.mdpi.com/article/10.3390/microorganisms14061343/s1, Dataset S1: Statistics on viral metagenomics obtained and phylogenetic analyzed contigs.

## Abbreviations

The following abbreviations are used in this manuscript:

CDC	Centers for Disease Control and Prevention
DNBs	DNA Nanoballs
NGS	Next-generation sequencing
ORF	Open Reading Frame
RdRp	RNA-dependent RNA polymerase
