# Supplementary material for: Metagenomic Analysis Reveals Viral Diversity in Phlebotomine Sand Flies from Caribbean Region in Colombia
Source: Microorganisms. 2026 Jun 15;14(6):1343. doi: 10.3390/microorganisms14061343 (PMC13304120; doi:10.3390/microorganisms14061343)
Supplement: Supplementary file 1 [file microorganisms-14-01343-s001.zip › Dataset_S1.pdf]

# Dataset S1: Statistics on viral metagenomics obtained and phylogenetic analyzed contigs

Only contigs longer than 300 bp with significant similarity to viral proteins (e-value <1e−5) were retained.

Although the amino acid identity values relative to currently available reference sequences were low, several lines of evidence support the viral nature of these sequences, including conserved genome organization, expected ORF architecture, and the presence of characteristic viral domains. Such low similarity values are not unexpected in metagenomic studies and may reflect the high genetic diversity and underrepresentation of insect-associated viruses in current databases.

- ***Dicistoviridae* (Sample: Pi.evansi_Co):** Total contigs=410; MinLength=200; MaxLength=10.191; Assigned contigs=243 (Cellular organisms=229 and Viruses=14); mode=BlastX; N50=509

- ***Phenuiviridae* (Sample: Lu.gomezi_Ce65):** Total contigs =28.004; MinLength=300; MaxLength=15.798; Assigned=23.057 (Cellular organisms=23.009 and Viruses=20); mode=BlastX; N50=1.270

- ***Rhabdoviridae* (Sample: Pi.evansi_Ce64):** Total contigs=3.522; MinLength=300; MaxLength=14.240; Assigned=2.945 (Cellular organisms=2.931 and Viruses=10); mode=BlastX; N50=541

➢

| Sample | Total reads | Contig name | NCBI ID | Contig size | Mean Coverage | Mapped reads | RPM | Virus family |
|---|---|---|---|---|---|---|---|---|
| Pi.evansi_Co | 23.176.542 | Genome-Like_Dici | **SRR36473596** | 10.191 | 797 | 8.285 | 357,5 | *Dicistoviridae* |
| | | DicistroV6000 | **SRR36473595** | 6.137 | 625 | 13.266 | 572,4 | |
| | | DicistroV3000 | **SRR36473594** | 3.158 | 313 | 3.581 | 154,5 | |
| Lu.gomezi_Ce65 | 78.547.734 | PhenuiMV93 | **SRR33224308** | 3.151 | 533,9 | 7.436 | 94,7 | *Phenuiviridae* |
| | | PhenuiV93 | **SRR33224307** | 6.648 | 563,1 | 13.608 | 173,3 | |
| Pi.evansi_Ce64 | 39.194.694 | RhabdoV102 | **SRR36473592** | 14.240 | 61.898 | 314.917 | 8.034,7 | *Rhabdoviridae* |
